# Data on patient-reported outcomes and the risk of readmission following a cardiac diagnosis

**DOI:** 10.1016/j.dib.2019.104859

**Published:** 2019-11-22

**Authors:** Britt Borregaard, Anne Vinggaard Christensen, Ola Ekholm, Trine Bernholdt Rasmussen, Knud Juel, Astrid Lauberg, Marianne Vámosi, Lars Thrysoee, Selina Kikkenborg Berg

**Affiliations:** a Department of Cardiothoracic and Vascular Surgery, Odense University Hospital, Odense, Denmark; b University of Southern Denmark, Odense, Denmark; c Department of Cardiology, Rigshospitalet, Copenhagen University Hospital, Copenhagen, Denmark; d National Institute of Public Health, University of Southern Denmark, Copenhagen, Denmark; e Department of Cardiology, Herlev and Gentofte University Hospital, Denmark; f Department of Cardiology and Cardiothoracic Surgery, Aalborg University Hospital, Aalborg, Denmark; g Department of Public Health, Section for Nursing, Aarhus University, Aarhus & Department of Cardiology, Aarhus University Hospital, Aarhus, Denmark; h Department of Cardiology, Odense University Hospital, Odense, Denmark; i Institute of Clinical Medicine, University of Copenhagen, Copenhagen, Denmark

**Keywords:** Cardiac readmission, Patient-reported outcomes, Anxiety, Depression, Quality of life, Cardiology

## Abstract

The data presented in this paper describe a supplementary figure and supplementary tables to the research article; Patient-reported outcomes predict high readmission rates among patients with cardiac diagnoses - Findings from the DenHeart study [1]. The data reports on findings from the DenHeart study, investigating the association between patient-reported outcomes (PROs) and the risk of readmission after a cardiac diagnosis. Data from a national survey with register-based follow-up of a cohort of 34,564 patients were analysed. PROs included the following instruments; The Short Form-12 (SF-12), the Hospital Anxiety and Depression Scale (HADS), the EuroQol 5 Dimensions 5 Levels (EQ-5D 5L), the HeartQol and the Edmonton Symptom Assessment Scale (ESAS). The included tables show the association between PROs and the risk of readmission and the figure illustrates the cumulative incidence function of readmission.

**Table:** Specifications Table

Subject	*Health science*
Specific subject area	*Cardiology and Cardiovascular Medicine*
Type of data	*Tables, figure*
How data were acquired	*Survey data (validated, licensed patient-reported outcome measures)* *Register-based data*
Data format	*Analysed*
Parameters for data collection	*The survey data included the following patient-reported outcome measures: Included the validated standardised questionnaires Short Form-12 (SF-12), the Hospital Anxiety and Depression Scale (HADS), the EuroQol 5 Dimensions 5 Levels (EQ-5D 5L), the HeartQol and the Edmonton Symptom Assessment Scale (ESAS)* *Clinical and socio-demographic data were register-based and obtained from the Danish National Patient Register, the Danish Civil Registration System and the Danish Education Register.*
Description of data collection	*A national survey, including six validated patient-reported outcome measurements and register-based follow-up data. The questionnaire was handed out at discharge from one of the five Danish Heart Centres from April 2013 to April 2014.*
Data source location	*Statistics Denmark, Copenhagen, Denmark*
Data accessibility	*The raw data (anonymised) can become available by request to the DenHeart-publication committee (*www.denheart.dk*) and after approval by the Danish Data Protection Agency. Potential access to data will be provided through Statistics Denmark.*
Related research article	Vámosi M, Lauberg A, Borregaard B, Christensen AV, Thrysoee L, Rasmussen TB, Ekholm O, Juel K, Berg SK. Patient-reported outcomes predict high readmission rates among patients with cardiac diagnoses Findings from the DenHeart study. International Journal of Cardiology. DOI: https://doi.org/10.1016/j.ijcard.2019.09.046.

**Table:** 

**Value of the Data** • Patient-reported outcomes (PROs) are known to be associated with worse outcomes following several cardiac diseases. Thus, data in the current paper provide information on the association between PROs at discharge and the risk of readmission among patients with different cardiac diagnoses. • Detailed data on the risk of readmission among patients with arrhythmia, heart failure, congenital heart disease, infectious heart disease, heart valve disease, heart transplant patients and other heart diagnoses are reported. • The data can inform health care professionals on the risk of readmission among different cardiac diagnostic groups. This knowledge can inform future treatment and care, as PROs may be used as risk assessment tools in clinical practice.

## 1 Data

**Fig. 1:**
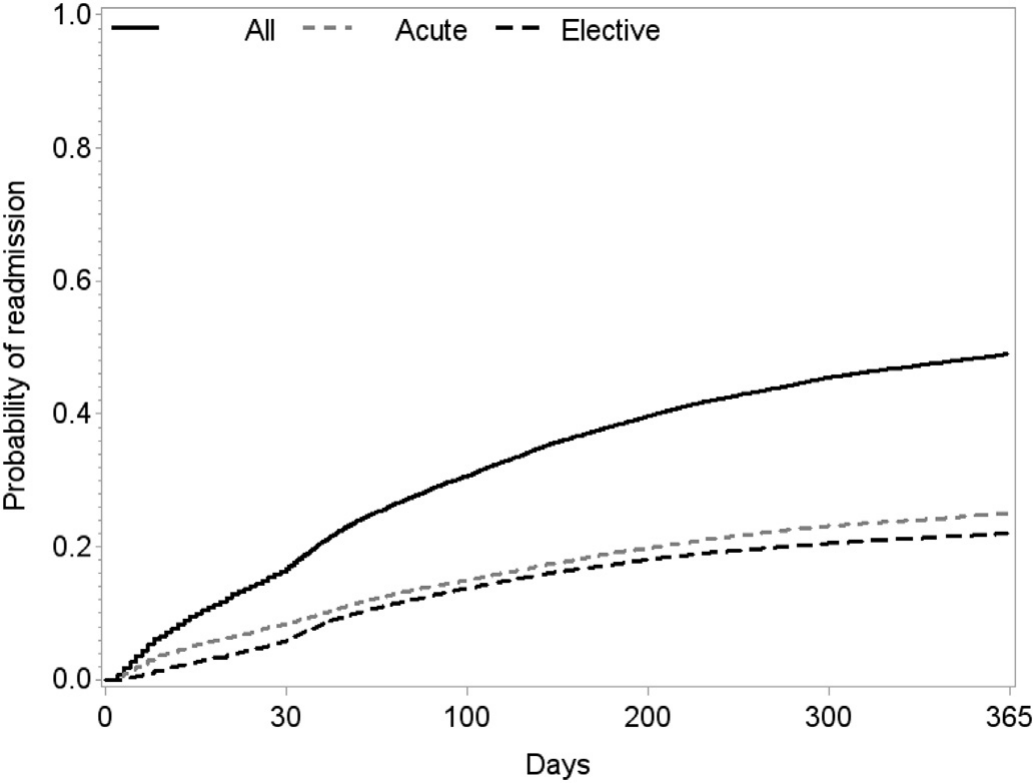
The cumulative incidence function of readmission. The Figure illustrates the cumulative incidence function of readmission with death as a possible competing risk. All-cause readmission, acute and elective cardiac readmission are illustrated.

The data shared in this paper are based on the DenHeart study [[1], [2]]; a national survey with register-based follow-up conducted in Denmark from April 2013 to April 2014. All cardiac patients who were discharged or transferred from one of the five Danish Heart Centres were invited to complete a paper-based questionnaire at discharge. Table 1 outlines the included patient-reported outcome measures of the survey. Table 2 shows the association between patient-reported outcomes (PROs) and cardiac readmissions within one year after discharge, whereas Table 3 shows the association between PROs and acute cardiac readmissions within 30 days after discharge among all patients and patients diagnosed with ischemic heart disease and arrhythmia, respectively. The cumulative incidence functions of all-cause readmissions and acute and elective cardiac readmissions, respectively, with death as a possible competing risk are illustrated in Fig. 1.

**Table 1:** Included patient-reported outcome measures of the DenHeart survey.

Patient-reported outcome measures
The Short Form-12 (**SF-12**) A 12-item brief generic instrument measuring overall health status. Two summary scores: the Physical Component Summary (PCS) and the Mental Component Summary (MCS) [7].
The Hospital Anxiety and Depression Scale (**HADS**) A 14-item disease-specific instrument measuring symptom of anxiety and depression. Subscales: HADS-A (anxiety) and HADS-D (depression) [8].
The EuroQol 5 Dimensions 5 levels (**EQ-5D 5L**) A standardised measure of health status (generic). The EQ-5D 5L consists of a classification system, the EQ-5D Index Score (5 items), and a Visual Analogue Scale, the EQ-5D VAS (0-100) [9].
The **HeartQol** A 14-item, disease-specific questionnaire measuring health-related quality of life. Subscales: a global score and two subscales (a physical scale and an emotional scale) [10,11].
The Edmonton Symptom Assessment Scale (**ESAS**) A generic questionnaire measuring symptom burden, ranging from 0 to 10 [12].

**Table 2:** Various patient-reported outcomes and the association with cardiac readmissions within one year following hospital discharge.

	All patients	Ischemic heart disease	Arrhythmia	Heart failure	Heart valve disease	Observation for heart disease
	HR (Cl)^a^	HR (CI)^a^	HR (CI)^a^	HR (CI)^a^	HR (CI)^a^	HR (CI)^a^
HADS-A≥8 vs. HADS-A<8	1.24 (1.17–1.32)	1.27 (1.15–1.39)	1.30 (1.16–1.46)	1.14 (0.90–1.44)	1.02 (0.82–1.26)	1.21 (0.98–1.49)
HADS-D≥8 vs. HADS-D<8	1.30 (1.21–1.39)	1.36 (1.22–1.52)	1.41 (1.23–1.62)	1.15 (0.88–1.49)	0.84 (0.65–1.07)	1.40 (1.10–1.79)
SF-12 PCS						
Index score per 1 point	0.98 (0.98–0.98)	0.98 (0.98–0.98)	0.98 (0.98–0.99)	0.98 (0.96–0.99)	1.01 (1.00–1.02)	0.98 (0.97–0.99)
SF-12 MCS						
Percentiles						
<20	1.41 (1.28–1.55)	1.32 (1.13–1.53)	1.56 (1.30–1.87)	1.32 (0.90–1.95)	0.61 (0.42–0.87)	1.35 (0.95–1.90)
20–39	1.21 (1.10–1.34)	1.15 (0.98–1.35)	1.32 (1.10–1.59)	1.05 (0.71–1.57)	0.74 (0.52–1.08)	1.50 (1.07–2.10)
40–59	0.96 (0.87–1.06)	0.94 (0.81–1.11)	1.10 (0.91–1.32)	0.77 (0.52–1.13)	0.59 (0.41–0.84)	0.84 (0.58–1.22)
60–79	1.01 (0.91–1.11)	0.95 (0.81–1.12)	1.06 (0.88–1.28)	0.97 (0.67–1.41)	0.79 (0.56–1.11)	0.86 (0.60–1.25)
≥80	1 (ref)	1 (ref)	1 (ref)	1 (ref)	1 (ref)	1 (ref)
EQ-5D						
Index score per 0.1 point	0.91 (0.89–0.93)	0.87 (0.85–0.90)	0.93 (0.89–0.96)	0.91 (0.85–0.97)	1.09 (1.02–1.17)	0.91 (0.86–0.97)
HeartQoL						
Global						
Index score per 1 point	0.72 (0.70–0.75)	0.76 (0.71–0.80)	0.70 (0.66–0.76)	0.66 (0.57–0.77)	1.20 (1.05–1.38)	0.75 (0.65–0.86)
ESAS per 1 point	1.01 (1.01–1.01)	1.01 (1.01–1.02)	1.02 (1.01–1.02)	1.02 (1.01–1.02)	0.99 (0.99–1.00)	1.01 (1.01–1.02)

Hazard ratios with 95% confidence intervals.

HADS-A = Hospital Anxiety and Depression Scale – Anxiety, HADS-D = Hospital Anxiety and Depression Scale – Depression, HR = hazard ratio, CI = confidence interval, MCS = mental component summary, PCS = physical component summary, ESAS = Edmonton Symptom Assessment Scale.

a Cox proportional hazards model with age as the time scale adjusted for sex, marital status, education, smoking behaviour, alcohol intake, body mass index and the Tu comorbidity index.

**Table 3:** Various patient-reported outcomes and the association with acute cardiac readmissions within 30 days following hospital discharge.

	All patients	Ischemic heart disease	Arrhythmia
	HR (Cl)^a^	HR (CI)^a^	HR (CI)^a^
HADS-A≥8 vs. HADS-A<8	1.36 (1.19–1.55)	1.33 (1.07–1.65)	1.70 (1.23–1.61)
HADS-D≥8 vs. HADS-D<8	1.57 (1.36–1.81)	1.50 (1.18–1.91)	1.93 (1.46–2.53)
SF-12 PCS			
Index score per 1 point	0.98 (0.98–0.99)	0.98 (0.97–0.99)	0.99 (0.98–1.00)
SF-12 MCS			
Percentiles			
<20	1.61 (1.30–2.00)	1.06 (0.74–1.53)	2.18 (1.43–3.32)
20–39	1.19 (0.95–1.49)	0.90 (0.62–1.31)	1.80 (1.18–2.74)
40–59	1.13 (0.90–1.41)	0.91 (0.63–1.32)	1.13 (0.72–1.77)
60–79	0.94 (0.74–1.19)	0.70 (0.48–1.02)	1.06 (0.68–1.64)
≥80	1 (ref)	1 (ref)	1 (ref)
EQ-5D			
Index score per 0.1 point	0.89 (0.85–0.92)	0.87 (0.82–0.93)	0.93 (0.87–1.00)
HeartQoL			
Global			
Index score per 1 point	0.73 (0.68–0.80)	0.86 (0.75–0.99)	0.67 (0.58–0.78)
ESAS per 1 point	1.02 (1.01–1.02)	1.01 (1.01–1.02)	1.02 (1.01–1.03)

Hazard ratios with 95% confidence intervals.

HADS-A = Hospital Anxiety and Depression Scale – Anxiety, HADS-D = Hospital Anxiety and Depression Scale – Depression, HR = hazard ratio, CI = confidence interval, MCS = mental component summary, PCS = physical component summary, ESAS = Edmonton Symptom Assessment Scale.

a Cox proportional hazards model with age as the time scale adjusted for sex, marital status, education, smoking behaviour, alcohol intake, body mass index and the Tu comorbidity index.

## 2 Experimental design, materials, and methods

### 2.1 Population

Adult patients were consecutively included. Exclusion criteria were; patients who were below the age of 18 years, patients without a Danish civil registration number (due to lack of possibility of register-based follow-up) and patients who did not understand Danish.

The included patients were grouped based on their ICD-10 diagnosis (primary diagnosis):

- ***Ischemic heart disease***: I200-I259, T823D, Z951, Z955.
- ***Arrhythmia:*** I440-I459, I470-I499, Z950, I460, I469, R000, R001, R002, R008A, T750, T754, T821, T828.
- ***Heart failure:*** I500-I509, I420-I438, I110, I517, R570.
- ***Congenital heart disease:*** Q00-Q99, I278A, I279, I280.
- ***Infectious heart disease:*** I109, I300-I320, I330-I339, I389-I390, I400-I418, I328, I398, I514, T826, T827.
- ***Heart valve disease:*** I050-I060, I340-I372, Z952-Z954, I391, I392, I511A.
- ***Heart transplant patients and other heart diagnoses:*** D151, E780, E785, I119, I260, I270, I272, I278, I279A, I510, I513B, I518, I519, I710-I714, I716, I719, J819, J960, J969, S250, S260, S268, S269, Z958, Z959, T862-T863, Z941, Z943.

### 2.2 Variables

#### 2.2.1 Clinical and sociodemographic data

Clinical and sociodemographic data were obtained from the following national registers: The Danish Civil Registration System [3], the Danish National Patient Register [4] and the Danish Education Register [5]. Data on comorbidities were used to calculate the Tu comorbidity index score, which includes several cardiac-related co-morbidities based on primary and secondary diagnoses [6].

Information on smoking, alcohol consumption, height and weight for calculating body mass index (BMI) were self-reported data and obtained through the survey. These data were used as potential confounders in the current analyses.

#### 2.2.2 Patient-reported outcomes

The following PRO measurements were included in the survey:

- The Short Form-12 (**SF-12**): The SF-12 is a brief self-reported measure of overall health status. The scores are expressed in terms of two summary scores: the Physical Component Summary (PCS) and the Mental Component Summary (MCS). A higher score indicates a better health status [7].
- The Hospital Anxiety and Depression Scale (**HADS**): The HADS is composed of 14 items (seven items to assess anxiety, HADS-A, and seven to assess depression, HADS-D). The subscales range from 0 (minimum level) to 21 (maximum level), and the cut-off score ≥ 8 suggests the presence of anxiety or depression [8].
- The EuroQol 5 Dimensions 5 Levels (**EQ-5D 5L**): The EQ-5D 5L is a standardised measure of health status. The EQ-5D 5L consists of a classification system, the EQ-5D Index Score, and a Visual Analogue Scale, the EQ-5D VAS. Higher scores indicate a better self-perceived health [9].
- The **HeartQol**: The HeartQol is a 14-item, disease-specific questionnaire measuring health-related quality of life in cardiac patients. The HeartQol is expressed in a global score and two subscales (a physical scale and an emotional scale). The scales range from 0 to 3, with better scores indicating a better self-rated quality of life [10,11].
- The Edmonton Symptom Assessment Scale (**ESAS**): The ESAS assess symptoms, including pain, tiredness, nausea, depression, anxiety, drowsiness, appetite, well-being and shortness of breath. The scores of ESAS range from 0 to 10, with lower scores indicating better status [12].

#### 2.2.3 Readmission and mortality

Readmissions were defined as admissions occurring more than 24 hours after the index admission and were obtained from the Danish National Patient Register [4]. Readmissions up to one year after the index admission were included. In this dataset, we included both planned (elective) and unplanned (acute) readmissions, which were pre-defined based on data from the registry. Similarly, causes of readmissions were based on the ICD-10 coding.

Data on all-cause mortality were obtained from the Danish Civil Registration System [3].

### 2.3 Statistical analyses

To investigate the association between PROs and the risk of readmission, multivariable Cox proportional hazard models were performed with age as the underlying time scale. Results were reported as hazard ratios (HR) with 95% confidence intervals (CI). The models were adjusted for potential confounders (sex, marital status, educational level, smoking, alcohol intake, body mass index (BMI) and co-morbidity). The models were performed for the overall population and stratified by diagnostic groups, Table 2, Table 3 The proportional hazard assumption and linear effects were checked graphically, and the assumption was met for all continuous scores, except the SF-12 MCS, which therefore was divided into quintiles for this score. The HADS-subscales were included as dichotomous variables (≥8 vs < 8).

To account for death as a competing risk, the incidence of readmission was evaluated using a cumulative incidence function in a Fine and Gray Proportional Hazard Model [13], Fig. 1.

SAS version 9.4 was used for the analyses.

## Funding

The study was funded by Rigshospitalet, Herlev-Gentofte Hospital, Odense University Hospital, Aarhus University Hospital, Aalborg University Hospital and the Novo Nordisk Foundation (grant number: 7229).
